# Non-Coding RNAs and their Integrated Networks

**DOI:** 10.1515/jib-2019-0027

**Published:** 2019-07-13

**Authors:** Peijing Zhang, Wenyi Wu, Qi Chen, Ming Chen

**Affiliations:** Department of Bioinformatics, State Key Laboratory of Plant Physiology and Biochemistry, College of Life Sciences, Zhejiang University, Hangzhou 310058, China; James D. Watson Institute of Genome Sciences, Zhejiang University, Hangzhou 310058, China

**Keywords:** non-coding RNA, integrated network, regulatory ncRNA, ncRNA interaction, ceRNA network

## Abstract

Eukaryotic genomes are pervasively transcribed. Besides protein-coding RNAs, there are different types of non-coding RNAs that modulate complex molecular and cellular processes. RNA sequencing technologies and bioinformatics methods greatly promoted the study of ncRNAs, which revealed ncRNAs’ essential roles in diverse aspects of biological functions. As important key players in gene regulatory networks, ncRNAs work with other biomolecules, including coding and non-coding RNAs, DNAs and proteins. In this review, we discuss the distinct types of ncRNAs, including housekeeping ncRNAs and regulatory ncRNAs, their versatile functions and interactions, transcription, translation, and modification. Moreover, we summarize the integrated networks of ncRNA interactions, providing a comprehensive landscape of ncRNAs regulatory roles.

## 1 Introduction

As a template for protein synthesis, messenger RNAs (mRNAs) have become the major research focus for a long time, while non-coding RNAs (ncRNAs) were considered as by-products of massive transcription with less biological meaning. Since the discovery of ribosomal RNA (rRNA) and transfer RNA (tRNA) in the late 1950s, varieties of RNA species have gradually surfaced, which revealed an unsuspected non-coding world. The combination of large-scale sequencing and computational analysis greatly helps to understand the RNA world. At the beginning of the 21^st^ century, initial sequencing and analysis of human [1] and mouse genome [2] revealed that 98% of the “junk” DNAs can be transcribed. Besides mRNAs that have already annotated, most transcripts do not seem to encode proteins. Therefore, those transcripts are commonly referred to as ncRNAs [3]. Soon afterwards, the Human Genome Project (HGP) was achieved in 2005 [4] and abundant long non-coding RNAs (lncRNAs) were detected in mammals [5], [6]. Later, the widespread application of high throughput sequencing further allowed for more accurate profiling of ncRNAs [7], [8]. The ENCODE (Encyclopedia of DNA Elements) project that launched in 2005 and its recent reports revealed that up to 80% of the human genome has the capacity to transcribe into ncRNAs [9], [10]. The large scale datasets generated from those projects have facilitated the establishment of many public databases, including Rfam [11], NONCODE [12], miRbase [13], and circBase [14].

In general, ncRNAs are found to participate in multiple biological processes, regulate physiological and developmental processes or even disease. They have been identified as tumor suppressors and oncogenic drivers in various cancer types [15]. In plants, ncRNAs have emerged as key regulatory molecules in stress responses [16]. Accumulating evidence indicates that ncRNA analysis has become a frontier research trend, current signs of progress have yielded novel insights into them. With the development of experimental techniques and big data analysis, diverse interactions of ncRNAs have been characterized gradually and form interconnected complex networks. The overall ncRNA network consists of various types of molecules from multi-omics datasets, involved in different subcellular localizations. Considering the rapid growth of ncRNA field, a comprehensive review of the current advances in ncRNAs that involves varieties of ncRNA species and their interrelationship is urgent. In this review, we briefly describe ncRNA transcription and the non-coding members, and then summarize three layers of ncRNA related interactions, which is a help to illustrate those complex ncRNA integrated networks.

## 2 Various ncRNA Species in Eukaryotes

**Figure 1:**
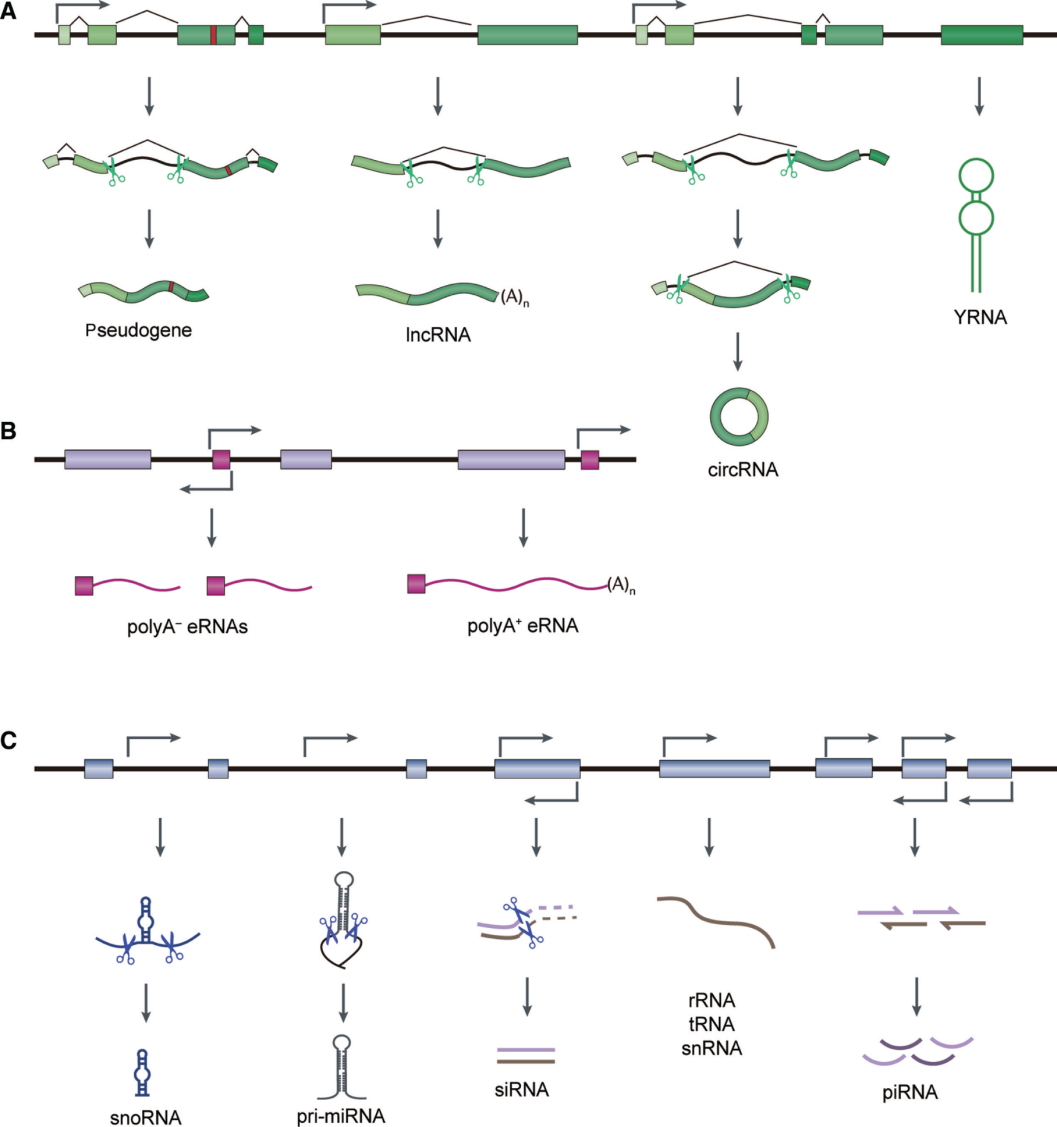
Distinct type of ncRNAs are transcribed from eukaryotic genomes. (A) Exons in protein-coding genes can be transcribed into pseudogenes, lncRNAs, and circRNAs, most of which have introns discarded. Y RNAs transcribed from individual genes. (B) Enhancer regions can be transcribed into divergent transcripts, known as eRNAs. (C) Intronic sequences can encode for snoRNAs or miRNAs. TEs can be transcribed into siRNA. Genes for rRNAs, tRNAs, or snRNAs are transcribed from separate genes. piRNAs and miRNAs can also be derived from various intergenic regions.

Eukaryotic transcription from different genomic regions and RNA processing produce various ncRNA species. Several parts of DNAs could be transcribed into ncRNAs, such as protein-coding genes, enhancer regions or transposon elements (Figure 1). Considering their regulatory roles, ncRNA could be divided into two categories (Table 1). Housekeeping ncRNAs are abundantly and ubiquitously expressed in cells, primarily regulate generic cellular functions. While regulatory ncRNAs are usually considered as key regulatory RNA molecules, function as regulators of gene expression at epigenetic, transcriptional, and post-transcriptional levels [17], [18], [19].

**Table 1:** Classification of ncRNAs.

Type	Abbreviation	Full name	Size
Housekeeping ncRNAs	rRNA	ribosomal RNA	120–4,500 nt
	tRNA	tansfer RNA	76–90 nt
	snRNA	small nuclear RNA	100–300 nt
	snoRNA	small nucleolar RNA	60–400
	TERC	telomerase RNA	/
	tRF	tRNA-Derived Fragments	16–28 nt
	tiRNA	tRNA halves	29–50 nt
Regulatory ncRNAs	miRNA	microRNA	21–23 nt
	siRNA	small interfering RNA	20–25 nt
	piRNA	piwi-interacting RNA	26–32 nt
	eRNA	enhancer RNA	50–2,000 nt
	lncRNA	long non-coding RNAs	>200 nt
	circRNA	circular RNA	100–10,000 nt
	Y RNA	Y RNA	/

### 2.1 Housekeeping ncRNAs

As early discovered ncRNA species, housekeeping ncRNAs have been intensively studied in the past decades, including rRNAs, tRNAs, small nuclear RNAs (snRNAs), small nucleolar RNAs (snoRNAs) and telomerase RNAs [20], [21]. These ncRNAs are usually small, ranging from 50 nucleotides (nt) to 500 nt, constitutively expressed in all cell types and necessary for cell viability [22]. Besides essential roles like rRNAs and tRNAs in protein synthesis, snRNAs in RNA splicing and snoRNAs in RNA modifications, some housekeeping ncRNAs could perform regulatory roles through cleavage. tRNA-derived RNA fragments (tRFs) and translation interfering tRNAs (tiRNAs) are new classes of small regulatory ncRNAs that are derived from tRNA or pre-tRNA [23]. Studies have suggested that tiRNAs could inhibit translation through recruitment of innovative packed aggregates of proteins and RNAs under stress situation [24], [25]. Additionally, some small RNAs derived from snoRNAs have been discovered by deep sequencing combing with bioinformatics analysis, such as sno-derived RNAs [26], sno-miRNAs [27], and sno-piRNAs [28].

### 2.2 Regulatory ncRNAs

Based on their average size, regulatory ncRNAs could be further classified as small non-coding RNAs (sncRNAs) with comprising transcripts fewer than 200 nt, and lncRNAs with greater than 200 nt. The main classes of small ncRNAs are microRNA (miRNA), small interfering RNAs (siRNAs), piwi-interacting RNAs (piRNAs). However, some ncRNAs with variable length might belong to two classifications at the same time, such as promoter-associated transcripts (PATs), enhancer RNAs (eRNAs), and circular RNAs (circRNAs).

miRNAs are the most abundant class of small ncRNAs that are generated from transcribed hairpin loop structures [29], [30], [31]. They regulate gene expression in both cytoplasm and nucleus through different mechanisms and mediate gene silencing at the post-transcriptional level [29]. As key players in the complex interplay among diverse RNA species, miRNAs have been considered research hotspot for several years. The latest miRBase release (v22) adds 48 new species, now contains 38,589 hairpin precursors and 48,860 mature miRNAs from 271 organisms, including animals, plants, unicellular algae, and viruses [32].

siRNAs are a class of double-stranded RNA molecules and play essential roles in RNA interference (RNAi) pathway. Based on their origins, siRNAs can be divided into two subcategories, exogenous siRNA (exo-siRNA), products of exogenous nucleic acid resulting from artificial insertion or virus infections, and endogenous siRNAs (endo-siRNA) that are arising from endogenous genomic locus and mostly transcribed from transposon elements (TEs) [33], [34]. In plants, the group of siRNAs is very diverse and contains subcategories, including trans-acting siRNAs (tas-siRNAs), heterochromatic siRNAs (hc-siRNAs) [35], repeat-associated siRNAs (ra-siRNAs), long siRNAs (lsiRNAs) [36], and natural antisense siRNAs (nat-siRNAs) that are also identified in animals [37].

Named after PIWI proteins, piRNAs are a class of animal-specific small ncRNAs. Mature piRNAs are derived from two major pathways, precursors are transcribed from piRNA clusters and cleaved by PIWI proteins in the primary pathway, and then modified through “ping-pong” cycle in the second [38], [39]. Distinct from miRNAs and siRNAs, piRNAs are processed in a Dicer-independent manner from single-stranded RNA precursors [40] and they only function through binding with PIWI proteins [41]. piRNAs are associated with PIWI proteins and form piRNA-induced silencing complexes, silence their targets at transcriptional and post-transcriptional levels [42], which acts in a similar manner that endo-siRNAs silence TEs in plants. Endo-siRNAs also silence TEs in animals, but the piRNA pathway is at the forefront of defense against transposons in germ cells [43].

LncRNAs are defined as transcripts that longer than 200 nt and lack of protein-coding ability. Based on their location with respect to protein-coding genes, lncRNA can be divided into five subcategories [44]. Long intergenic ncRNAs (lincRNAs) are transcribed from both strands in intergenic regions. Long intronic ncRNAs are completely transcribed from introns of protein-coding genes. Sense lncRNAs are transcribed from the sense strand and containing exons of protein-coding genes. In contrast to sense lncRNAs, antisense lncRNAs are transcribed from the antisense strand, overlapping with exonic or intronic regions, or covering the protein-coding sequence through an intron, while bidirectional lncRNAs, localized in the proximity of a coding transcript on the opposite strand [45]. According to their regulatory effects on DNA sequences, lncRNA could be classified as cis-lncRNAs (cis-acting lncRNAs) that regulate the expression of genes in close proximity, and trans-lncRNAs (trans-acting lncRNAs) that regulate distant genes [46]. Some lncRNAs could be further processed to generate small ncRNAs, such as miRNAs, piRNAs, and snoRNAs [47].

eRNAs are emerging as a group of ncRNAs transcribed from transcriptional enhancers. Besides similar length and protein-coding potentiality, eRNAs share some transcriptional features with lncRNAs, like having both sense and antisense transcripts by bidirectional transcription [48], [49], [50]. Bidirectional transcription of enhancer regions generates comparatively shorter (0.5–2kb) and non-polyadenylated eRNAs (polyA-eRNAs) [51], while long (>4kb) and polyadenylated eRNAs (polyA + eRNAs) are usually unidirectional transcribed from enhancers [52] (Figure 1). As for eRNAs that are generated by transcription initiation at enhancers in intragenic regions, those are multiexonic polyA + eRNAs (meRNAs) [50], [53]. Although having similar properties, eRNAs are 5′ capped, unstable, exhibiting short half-lives and easily degraded by the exosome complex, which was quite different from lncRNAs [54], [55], [56], [57], [58], [59].

CircRNAs are a unique class of endogenous ncRNAs that form covalently closed loop structures. Although discovered for decades, advanced deep sequencing and bioinformatics analysis made circRNA gain more attention recently [60]. CircRNAs have a wide range of sizes, ranging from 100 nt to over 10,000 nt, while most of them in mammals and plants are hundreds of nucleotides [61], [62]. CircRNAs are the circularization product from splicing events, and generate from exons, introns, intergenic regions, untranslated regions (UTRs) or even tRNAs [63]. Similar to other regulatory ncRNAs, circRNAs play important roles in diverse biological processes, such as regulating alternative RNA splicing or transcription [64], acting as competing endogenous RNAs (ceRNAs) [65] or miRNA sponges [66].

## 3 ncRNA Interactions

### 3.1 ncRNAs Interact with mRNAs

To define biological functions for ncRNAs, some experimental approaches are employed for the investigation of ncRNA-based targeting, like PAR-CLIP [67], HITS-CLIP [68], and CLASH [69]. HITS-CLIP is widely used for functional protein-RNA interaction sites identifying and miRNA-mRNA regulatory interactions analysis [70], [71]. Compared to the HITS-CLIP, PAR-CLIP improves RNA recovery efficiency and indicates the site of targeting more precisely through identifying the location of the crosslink [71], [72]. LIGR-seq (LIGation of interacting RNA followed by high-throughput sequencing) is a new developed method that enables the global-scale mapping of RNA-RNA duplexes *in vivo* [73], which enlarges the landscape of ncRNA-mRNA interactions. Those contain unexpected interactions between snoRNAs and mRNAs, such as the orphan C/D box snoRNA SNORD83B controls steady-state levels of target mRNAs. These detections may be additional examples for ncRNA-mediated gene regulation. Besides, the ANXA2 mRNA and the ANXA2 Pseudogene ANXA2P2 may have direct or indirect interaction to promote tumor cell invasion through remodeling the hepatocellular carcinoma (HCC) cells movement that could be a novel predictive biomarker for the HCC patients risk evaluation of its recurrence or metastasis [74].

Furthermore, miRNA-mRNA interactions would result in the silencing of mRNA expression. In neoplasms, upregulated miRNAs could act as oncogenes through silencing the mRNAs encoding tumor suppressor proteins [75]. ncRNAs can also promote the expression of mRNA. The circular RNAs hsa_circ_0032462, hsa_circ_0028173, hsa_circ_0005909 regulate the Cell adhesion molecule 1 (CADM1) gene which is a co-expression mRNA functioning as a miRNA sponge in human osteosarcoma [76]. Thus, these osteosarcoma-related miRNAs could provide a solution in the development of new therapies for human osteosarcoma [77].

### 3.2 ncRNA-ncRNA Interactions

ncRNA-ncRNA crosstalk indicates the intersecting non-coding world, and these interactions could impact numerous biological processes, such as epigenetic modifications, transcription, and translation; hence gain a new layer of genomic regulation. Those miRNA response elements (MREs) such as circRNAs, lncRNAs, and eRNA act as competitive endogenous RNAs (ceRNAs), which has abundant implications for gene regulation during multiple physiological and pathophysiological processes at the post-transcriptional level. For example, functional experiments suggested that the circular RNA hsa_circ_0001368 decelerates the growth of gastric cancer by regulating miR-6506-5p/FOXO3 axis by serving as a ceRNAs to sponge miR-6506-5p. Thus hsa_circ_0001368 plays a tumor-suppression role in gastric cancer and may be adapted to the gastric cancer therapy [78].

In addition to the interaction between different ncRNAs, such as ceRNAs mentioned above, there are interactions between the same kind of ncRNAs, such as miRNA-miRNA and lncRNA-lncRNA. The interactions among multiple miRNAs have enriched the complex mechanisms of post-transcriptional regulations [79]. In mammals, Krek et al. experimentally validated a combination of miR-375, miR-124, and let-7b which lead to synergistic target inhibition [80]. In addition, functional cooperation of miR-125a, miR-125b, and miR-205 can act together to suppress erbB2/erbB3 expression in breast cancer cells, which manifest a novel breast cancer treatment via miRNA-dependent or -independent mechanisms [81]. What’s more, evidence indicated that lncRNA-lncRNA synergistic networks play a vital role in oncogenesis and tumor suppressor pathways. For instance, lncRNAs contribute to the p53 biological network, act as p53-regulator lncRNAs and p53-effector lncRNAs, thus may promote the development of diagnostic methods and therapies focused on these non-coding molecules [82]. We could identify synergistic lncRNA pairs via combining analysis of genome-wide expression datasets and functional information [83].

### 3.3 ncRNAs Interact with DNAs

Many ncRNAs serve as direct regulators on chromatin, as exemplified by lncRNAs and tiRNAs. Recent progress in high-throughput RNA sequencing facilitates the detection of promoter-proximal small RNAs, including tiRNAs, promoter-associated small RNAs (PASRs) [84], and transcription start site-associated RNAs (TSSa RNAs) [85]. They are possibly associated with nucleosome positioning, chromatin marking, and transcriptional regulation, such as tiRNAs are abundant at transcription-initiation chromatin marks.

An RNA sequencing technology called global RNA interactions with DNA by deep sequencing (GRID-seq) has been reported to detect chromatin-RNA interactions, comprehensively [86]. Two highly expressed human lncRNA NEAT1 (nuclear-enriched abundant transcript 1) and MALAT1 (metastasis-associated lung adenocarcinoma transcript 1) were identified by GRID-seq which localize to hundreds of genomic sites in mammals (mostly overactive genes) [87]. NEAT1 and MALAT1 exhibit colocalization, although they display distinct gene body binding patterns at active chromatin. Moreover, the function of enhancer-associated miRNAs has been demonstrated to be able to up-regulate transcription through miRNA-promoter interaction. miR-24-1 has been associated with a function that activates eRNA expression, alters histone modification which results in p300 and RNA polymerase II’s increased enrichment at the enhancer locus [84]. Besides, the lncRNA KHPS1 would induce the synthesis of eRNA Sphk1 towards a triplex-driven recruitment mechanism, which enhances the expression of specific target genes [88]. In addition, the conserved Y RNAs were found to be key factors for the initiation of DNA replication, preferentially with replicated chromatin [85]. Non-coding human Y RNAs can act not only as new cancer biomarkers and also molecular targets for anti-proliferative prevention [89]. hY1 and hY3 RNAs have been found to result in a remarkable cytostatic suppression of cell proliferation.

### 3.4 ncRNAs Interact with Proteins

ncRNA-protein interactions have critical roles in all aspects of gene expression, therefore, plenty of approaches have been created for comprehensively analysis. Development of deep-sequencing methods together with immunoprecipitation of RNA binding proteins (RBPs) has manifested a great diversity of ncRNA-associated proteins [90].

Housekeeping ncRNAs interacting with proteins can shape into varied ribonucleoprotein (RNP) complexes that perform various functions. For instance, snRNAs together with multiple proteins form spliceosomes (snRNPs) that participate in canonical splicing and alternative splicing. Many nucleotides in the pre-rRNA, pre-snRNA, and pre-tRNA undergo post-transcriptional modifications via small nucleolar RNP particles [49]. What’s more, interactions between regulatory RNAs and proteins are vital in interceding fundamental cellular processes. Small ncRNAs, like miRNAs, siRNAs, and piRNAs, are well known to affect RNA stability and translation during RNA interference pathway towards interacting with the Argonaute family proteins during RNA interference pathway. LncRNAs achieve their functions as RNPs in several ways like recruitment, inhibition, and act indirectly through genome organization and transcription [91]. There is a well-studied lncRNA Xist which is required during development in female mammals. Three proteins: SHARP, SAF-A, and LBR -interact with Xist are required for transcriptional silencing [92]. Moreover, circRNAs can serve as protein sponges translocate proteins to a specific subcellular location. The expression of circRNA-Foxo3 has significantly increased during cancer cell apoptosis, which is experimentally explored the possibility of inhibiting tumor growth with the delivery of circ-Foxo3 plasmid [93]. It has been clarified that protein recruitment by lncRNAs and circRNAs modifies the cellular concentration and localization of proteins.

## 4 Integrated ncRNA Networks

More and more evidence proves that ncRNAs provide diverse regulatory functions in the cell. Those RNA mediated interactions are often interconnected, thus, constructing complex regulatory RNA networks. Most ncRNAs, especially housekeeping ncRNAs, have been characterized through their functions in the nucleus and their interactions with various types of nuclear machinery may contribute to their nuclear retention [94]. However, many ncRNAs are also detected in the cytoplasm and play regulatory functions there. The ceRNA network is one of the most representative networks of regulatory ncRNA networks in transcriptomics, while those networks also involve crucial interphases between genomics and proteomics.

### 4.1 ceRNA Network

**Figure 2:**
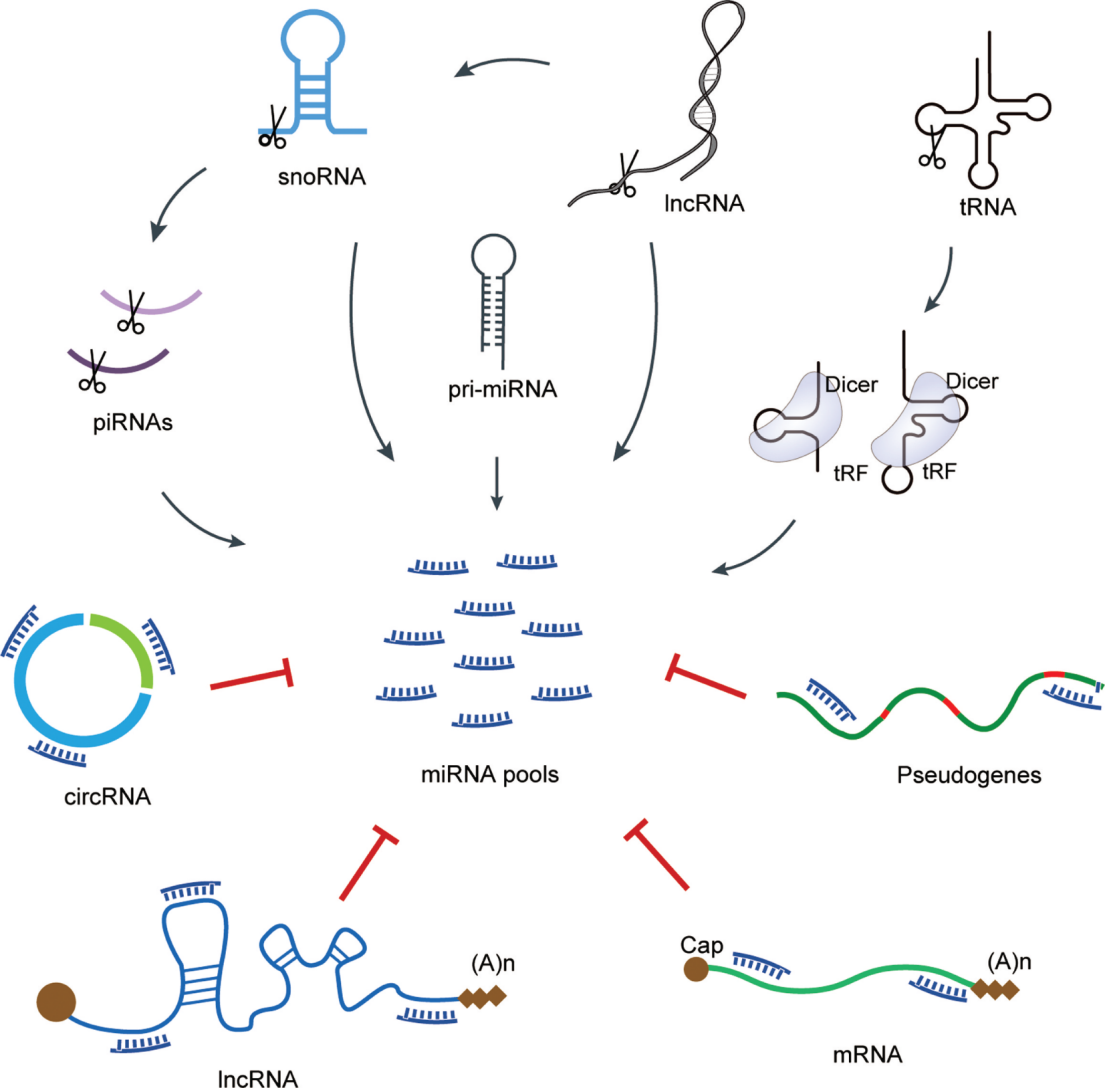
Network of miRNAs biogenesis and ceRNAs. miRNAs may be generated from lncRNAs, snRNAs, piRNAs or tRNAs, that repress protein-coding mRNAs by binding to MREs. Different classes of ceRNAs, such as circRNAs, lncRNAs, and pseudogenes, also contain MREs and compete for miRNA binding.

Based on the ceRNA hypothesis, the cross-talk between RNAs through MREs could form a large-scale regulatory network across the transcriptome, including both coding and non-coding RNAs [95]. Like other ceRNAs namely pseudogenes, lncRNAs and circRNAs, mRNAs also contain MREs, which are normally located within the 3′UTR. All of these RNAs potentially compete for miRNA binding, thereby regulating miRNA repression. Thus, mRNAs, different types of ceRNAs and miRNAs would form a network of interactions, as called ceRNA network (Figure 2). ceRNAs usually contain carry MREs for multiple miRNAs, each miRNA can regulate multiple ceRNAs and most ceRNAs are regulated by more than one miRNA.

According to ceRNA theory, the existence of this network has three important conditions [95]. First, the relative concentration of ceRNAs and their miRNAs would impact the expression of competing ceRNAs. Considering the density of ceRNA network connections, only the expression level of ceRNAs is large enough could overcome or relieve miRNAs’ repression on downstream mRNA targets. Second, the numbers of MRE normally influence the effectiveness of a ceRNA. Furthermore, in a given cell type or at a specific moment, the identity, concentration, and subcellular distribution of ceRNA and miRNA species impact ceRNA networks. Third, miRNA binding is impact by MREs, not all the MREs on ceRNAs are equal. The specific nucleotide composition of MREs might be partially different even if they are predicted to bind the same miRNA. The binding effectiveness of each MRE is critical for overall ceRNA function. Additionally, if a miRNA is sequestered by ceRNAs, its primary targets would be preferentially affected. Although hundreds of RNAs are predicted to be targeted by a miRNA, their degrees of repression are not same; only a few of them are primary targets while the rest are finely tuned [96], [97].

In ceRNA networks, the majority of validated ceRNAs are mRNAs. They compete for miRNA binding to sequester miRNAs from potential alternative targets, this biological role depends on their ability to compete for miRNA binding and are independent of protein-coding function [96]. Zinc finger E-box binding homeobox 2 (ZEB2) mRNA, has been validated as a ceRNA and regulates PTEN expression levels by sequestering several miRNAs, including miR-181, miR-200b, miR-25, and miR-92a. Attenuation of ZEB2 expression leads to inhibition of PTEN in human melanoma [98]. Additionally, 3′UTRs that contain MREs for multiple miRNAs, are critical for the function of mRNAs as ceRNAs. It has been reported that the versican (VCAN) 3′UTR binds to miR-144 and miR-136 to modulate PTEN level, and cell cycle regulator retinoblastoma 1 (Rb1) that targeted by miR-199a-3p and miR-144 could function as a ceRNA for VCAN [99]. Thus, VCAN 3′UTR binds and modulates miRNA activities as a miRNA sponge, and Rb1 and PTEN mRNAs are released for translation. CD34 and FN1 are additional validated VCAN ceRNAs, competing for binding to miR-133a, miR-144, miR-199a-3p, and miR-431 [100], [101]. As one of the most studied lncRNA, H19 is highly expressed in undifferentiated muscle cells and has canonical and non-canonical binding sites for the let-7 miRNA family. When the expression of H19 is reduced in differentiated cells, the expression of let-7 increases at the same time. H19 has been demonstrated competing with DICER and HMGA2 to modulate let-7 availability, as a molecular sponge [102]. Besides, H19 together with circRNA MYLK and CTDP1 compete for miRNA-29a-3p binding to regulated the expression of its target genes DNMT3B, HAS3, VEGFA, and ITGB1, which would result in cancer growth and metastasis [65]. Pbcas4, the pseudogene of breast carcinoma amplified sequence 4 (BCAS4), is a conserved ceRNA in mouse and human, whose transcripts compete with BCAS4 mRNAs for binding to the common miR-185 [103].

### 4.2 ncRNA-Mediated Networks in Genomics and Proteomics

**Figure 3:**
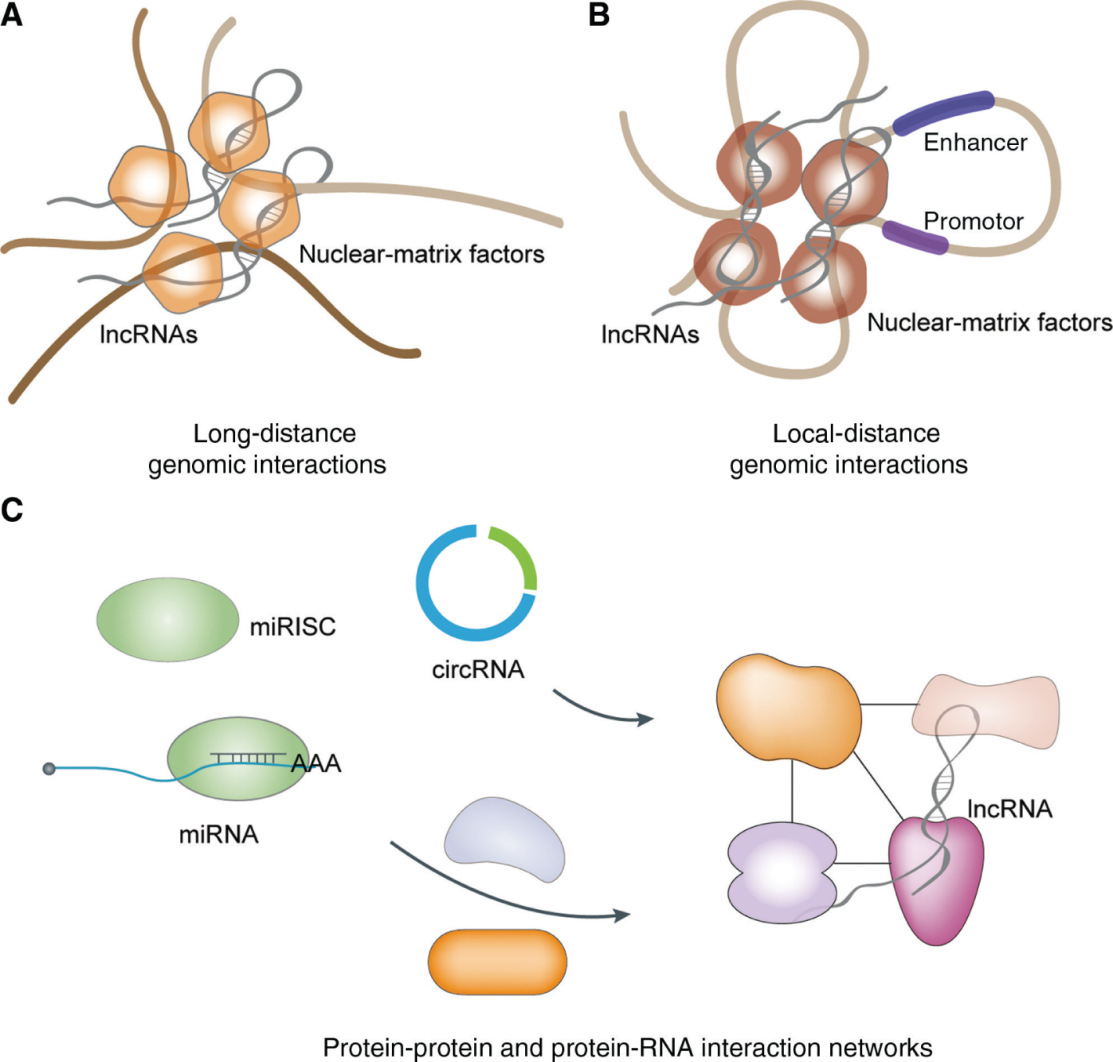
ncRNA-mediated networks are an integral part of genomics and proteomics. (A) ncRNAs regulate the organization of genome in the nucleus by interacting with nuclear-matrix factors. (B) ncRNAs mediate promoter-enhancer interactions to regulate the expression of protein-coding genes. (C) ncRNAs are extensively involved in protein interaction networks.

Besides ncRNA-ncRNA interactions, ncRNAs could regulate biological processes through diverse mechanisms. ncRNAs and their mediated networks are an integral part of genomics and proteomics (Figure 3).

ncRNAs could contribute to local and long-range genomic interactions (Figure 3). Functional studies of eRNAs and some lncRNAs have demonstrated that ncRNAs mediate promoter-enhancer interactions to regulate the expression of various protein-coding genes [59], [104], [105]. In the nucleus, ncRNAs play critical roles in the organization of the genome to coordinate the expression of gene clusters. Recent studies have shown that the Xist complex explores some larger genomic domains to help spread transcriptional repressor complexes during X-chromosome inactivation [106], [107]. This strategy could also be used to establish active and inhibitory domains that involve genomic segments separated by a long linear distance on the same chromosome or even from a different chromosome, which in turn may contribute to the organization of the genome in 3D space [108], [109].

At the transcriptional and the post-transcriptional levels, regulated gene expression determines the cell type-specific proteome, and ncRNAs are widely involved in protein-mediated interaction networks (Figure 3). There are numerous RNA-dependent protein-protein and protein-DNA interactions in the cell, ncRNAs play pivotal roles in those regulations in which RBPs, lncRNAs, miRNAs, or even circRNAs are involved [110], [111]. The positive feedforward of RBP HuR is a good example of protein-ncRNA interaction networks. HuR may associate with many mRNAs, which influence cell proliferation, survival, immunity, carcinogenesis, and stress responses [112], [113]. HuR and lincMD1 are both involved in muscle differentiation, and these two are under the inhibitory control of miR-133. Interestingly, HuR was identified as another component of the lincMD1-regulated circuitry. In that circuitry, HuR binds lincMD1 and protects it from Drosha cleavage at the expense of miR-133b biogenesis [51].

## 5 Discussion

Benefited from the rapid advance in sequencing technologies and bioinformatics approaches, different classes of ncRNAs have been detected and discovered in the past decades, leading to a complex ncRNA world. Sufficient evidence shows that ncRNA plays significant biological roles in regulation of cellular mechanisms, and is associated to higher eukaryotes complexity. ncRNAs can interact with different species of molecules, and regulate them through various molecular mechanisms generating a complex network. ncRNA-mediated networks not only contained interplay among RNAs, but also involved in genomics and proteomics, in both nucleus and cytoplasm.

The ceRNA hypothesis attributes potentially predictable function to the coding and noncoding transcriptome, which helps to systemically realize the ncRNA network. Although ceRNA networks are one of the most extensively studied ncRNA networks, novel ncRNA species will increase the complexity of these networks. Multiple types of molecules and biology pathways make it more difficult to comprehensively understand ncRNA functions in multi-level networks. Even though the accumulation of information about ncRNA interactions and their mediated networks is relatively slow, the development of tools in silico predictors and network analysis module may accelerate this process. ncRNA-mediated interactions could be captured by experiment detections, and those associations have been systematically analyzed and extended to more connections in various computation method. Based on those intricate interactions, network model could be constructed and predict potential ncRNA related associations.
